# Transferrin-Modified Triptolide Liposome Targeting Enhances Anti-Hepatocellular Carcinoma Effects

**DOI:** 10.3390/biomedicines11102869

**Published:** 2023-10-23

**Authors:** Xiaoli Zhao, Yifan Yang, Xuerong Su, Ying Xie, Yiyao Liang, Tong Zhou, Yangqian Wu, Liuqing Di

**Affiliations:** 1 College of Pharmacy, Nanjing University of Chinese Medicine, Nanjing 210023, China; leah_zhao@njucm.edu.cn (X.Z.); Yifan_Yang0801@163.com (Y.Y.); suxuer69@163.com (X.S.); xeeyeah@njucm.edu.cn (Y.X.); lyy541@126.com (Y.L.); 20210906@njucm.edu.cn (T.Z.); 20220988@njucm.edu.cn (Y.W.); 2 Jiangsu Provincial TCM Engineering Technology Research Center of High Efficient Drug Delivery System (DDS), Nanjing 210023, China

**Keywords:** targeted therapy, lipidosome, transferrin, anti-tumor, hepatocellular carcinoma, fluorescence imaging

## Abstract

Triptolide (TP) is an epoxy diterpene lactone compound isolated and purified from the traditional Chinese medicinal plant *Tripterygium wilfordii* Hook. f., which has been shown to inhibit the proliferation of hepatocellular carcinoma. However, due to problems with solubility, bioavailability, and adverse effects, the use and effectiveness of the drug are limited. In this study, a transferrin-modified TP liposome (TF-TP@LIP) was constructed for the delivery of TP. The thin-film hydration method was used to prepare TF-TP@LIP. The physicochemical properties, drug loading, particle size, polydispersity coefficient, and zeta potential of the liposomes were examined. The inhibitory effects of TF-TP@LIP on tumor cells in vitro were assessed using the HepG2 cell line. The biodistribution of TF-TP@LIP and its anti-tumor effects were investigated in tumor-bearing nude mice. The results showed that TF-TP@LIP was spherical, had a particle size of 130.33 ± 1.89 nm and zeta potential of −23.20 ± 0.90 mV, and was electronegative. Encapsulation and drug loading were 85.33 ± 0.41% and 9.96 ± 0.21%, respectively. The preparation was stable in serum over 24 h and showed biocompatibility and slow release of the drug. Flow cytometry and fluorescence microscopy showed that uptake of TF-TP@LIP was significantly higher than that of TP@LIP (*p* < 0.05), while MTT assays indicated mean median inhibition concentrations (IC_50_) of TP, TP@LIP, and TF-TP@ of 90.6 nM, 56.1 nM, and 42.3 nM, respectively, in HepG2 cell treated for 48 h. Real-time fluorescence imaging indicated a significant accumulation of DiR-labeled TF-TP@LIPs at tumor sites in nude mice, in contrast to DiR-only or DiR-labeled, indicating that modification with transferrin enhanced drug targeting to the tumor tissues. Compared with the TP and TP@LIP groups, the TF-TP@LIP group had a significant inhibitory effect on tumor growth. H&E staining results showed that TF-TP@LIP inhibited tumor growth and did not induce any significant pathological changes in the heart, liver, spleen, and kidneys of nude mice, with all liver and kidney indices within the normal range, with no significant differences compared with the control group, indicating the safety of the preparation. The findings indicated that modification by transferrin significantly enhanced the tumor-targeting ability of the liposomes and improved their anti-tumor effects in vivo. Reducing its distribution in normal tissues and decreasing its toxic effects suggest that the potential of TF-TP@LIP warrants further investigation for its clinical application.

## 1. Introduction

Hepatocellular carcinoma (HCC) is not only the most common form of primary liver cancer, but also the leading cause of cancer-related deaths worldwide. Its pathogenesis is closely related to chronic infection by the hepatitis B and C viruses, as well as alcoholic cirrhosis, and treatment options currently include surgical resection, liver transplantation, liver-directed therapy, and systemic chemotherapy. However, patients with liver cancer are usually diagnosed at an advanced stage, and as such, they cannot undergo surgical resection and liver transplantation [1]. In most countries, mortality from HCC is also almost equal to morbidity, largely due to poor response to both conventional chemotherapy and targeted therapeutic drugs [2,3,4]. Hence, in addition to early detection, diagnosis, and surgery, it is necessary to develop new treatment strategies to improve HCC treatment [5]. In this context, Chinese herbal medicine, one of the integrated treatment modalities, has gained increasing attention for its anti-inflammatory and anti-cancer properties.

Triptolide (TP), first isolated by Kup-chan et al. from *Tripterygium wilfordii* Hook. f. (TWHF) in 1972, has significant broad-spectrum anti-tumor and anti-autoimmune effects [6]. As the major active ingredient of TWHF, TP exhibits almost all these therapeutic effects, as well as, unfortunately, toxicity [7]. Recent studies have shown that TP displays significant anti-tumor activity and other potential therapeutic effects against various cancers, such as liver, lung, and pancreatic cancers [8,9], prompting extensive exploration of these properties [10,11]. In particular, TP has been reported to inhibit the proliferation and invasion of tumor cells with activities comparable to or even better than some traditional anti-tumor drugs, including adriamycin, mitomycin, paclitaxel, and cisplatin, to name a few [12]. The anti-tumor activity of TP is reflected in its strong cytotoxicity, which can eliminate drug resistance, while inhibiting neovascularization and tumor metastasis. Early studies have also shown that the anticancer properties of TP mostly involve its induction of apoptosis, thus preventing the development of many tumors [13,14]. However, the mechanism by which TP induces cell death varies depending on the cell type. In addition to apoptosis, TP can also affect the metabolism of tumor cells by reducing cell viability and growth through cell cycle arrest. However, as the clinical use of TP increased, several studies and clinical reports reported that TP also had serious adverse effects, including multi-organ toxicity (hepatotoxicity [15,16], nephrotoxicity [15], cardiotoxicity [17], and reproductive toxicity [18]). This, together with its poor water solubility, resulted in reduced clinical application of TP [19]. Consequently, it would be advantageous to design a drug delivery system that includes modification of the molecular structure, as well as providing effective delivery of TP to targeted sites, while reducing the accumulation of free drugs in other tissues and organs to lessen the incidence of toxic and adverse effects and improve therapeutic efficacy.

Considering the highly potent activity of TP against various types of malignant cells, the current study aimed to improve the treatment of HCC through the targeted delivery of TP to reduce its toxic side effects.

The phospholipid bilayers of liposomes increase the solubility of insoluble drugs, reduce the rate of blood clearance, and prolong the half-life of the drug in the body, thereby increasing bioavailability [20]. The advantages of using liposomes include increased circulation time, improved drug stability, lower toxicities, and enhanced targeting capability. However, it is worth noting that despite their efficacy in treating various cancers, liposomes lack the ability for specific targeting [21]. By introducing targeting molecules (e.g., peptides, monosaccharides, polysaccharides, folic acid, antibodies, or antibody fragments) on the liposome surfaces that can specifically bind to tumor cells, the retention of nano drugs within tumor tissues can be promoted, thus enhancing the efficiency of endocytosis and their enrichment within tumor cells [22]. Human transferrin receptors (TFRs) are single-chain transmembrane glycoproteins composed of 700 amino acids with two disulfide subunits that are involved in the transport of ferric ions [23,24]. Transferrin (TF) acts as a carrier of ferric ions and enters the cell via the TFR. To maintain their rapid proliferation, cancer cells require more iron than normal cells [25], and thus many tumor cells, such as HepG2 and MDA-MB 231 cells, overexpress the TFR. The elevated expression of the TFR can be used to enhance the targeting of therapeutic cargo-loaded nanoparticles, such as liposomes. Through receptor-mediated endocytosis (RME), the TFR-targeted nanocarriers can improve the specificity of the drug cargo towards cancer cells [26,27]. Many studies have used PEGylated liposomes combined with TF and PEG to achieve targetability and longevity for drug delivery to solid tumors [28,29]. To date, there have been several reports of the use of nano agents, including liposomes [30], cubosomes [31], polymer vesicles, and polymer nanoparticles [9,32] for targeting liver cancer.

In this study, a TF-mediated liposomal drug delivery system was designed to slow down its release in vivo, as well as increase its targeting ability in vivo and in vitro, thereby reducing the drug’s toxicity, while enhancing its effects against HCC.

## 2. Materials and Methods

### 2.1. Materials

Hydrogenated soy lecithin (HSPC), stearoyl phosphatidylethanolamine-polyethylene glycol 2000 (DSPE-PEG2000), and cholesterol (CHO) were purchased from A.V.T. Pharmaceutical Co., Ltd. (Shanghai, China). DSPE-PEG2000-MAL was obtained from the Xi’an Ruixi Biological Technology Co., Ltd. (Xi’an, China), while phosphate buffer (pH = 7.2–7.4) was obtained from Solarbio Science & Technology Co., Ltd. (Beijing, China). Holo-transferrin was obtained from Best Biological Technology Co., Ltd. (Nanjing, China), thiazolyl blue tetrazolium bromide (MTT) was obtained from Yuanye Bio-Technology Co., Ltd. (Shanghai, China), and fetal bovine serum (FBS) and Dulbecco’s modified Eagle medium (DMEM) were obtained from SenBeiJia Biological Technology Co., Ltd. (Nanjing, China). Triptolide (TP) was purchased from Nantong Feiyu Biological Technology Co., Ltd. (Nantong, China), and Traut’s reagent was purchased from Nanjing JinYibai Biological Technology Co., Ltd. (Nanjing, China), but obtained from the Labgic Technology Co., Ltd. (Beijing, China) together with 4-diamidino-2-phenylindole (DAPI, MW 350.2). Finally, coumarin-6 was purchased from Beyotime Biotechnology Co., Ltd. (Shanghai, China).

### 2.2. Cell Culture

The human hepatocellular carcinoma cell line (HepG2) was obtained from the Center for Excellence in Molecular Cell Science (Shanghai, China).

### 2.3. Animals

BALB/c nude mice (female, aged 6–8 weeks, 18–20 g) were purchased from Qinglongshan Laboratory Animal Company Limited (Nanjing, China). The Animal Care and Use Committee of Nanjing University of Chinese Medicine approved the animal experiments (approval number 202107A034). The animals were housed under pathogen-free conditions and provided with sterile food and water at Nanjing University of Chinese Medicine’s Laboratory Animal Center.

### 2.4. Preparation of Triptolide Liposomes (TP@LIP) and Triptolide Liposomes Modified by Transferrin (TF-TP@LIP)

TF-TP@LIP liposomes were prepared by membrane hydration. Using the encapsulation rate and drug loading as the main evaluation indices, we used a star design-response surface optimization method to optimize and verify the best prescription of TP@LIP. Briefly, this involved dissolving TF in PBS at a concentration of 10 mg/mL prior to thiolation with Traut’s reagent. The molar ratio of Traut’s reagent to TF was 20:1, and the reaction was allowed to proceed for 1 h on a shaker protected from light. DSPE-PEG2000-MAL was then dissolved in 2.5 mL of PBS (pH 6.5), and the mixture was allowed to react with the thiolated TF in darkness overnight to yield DSPE-PEG2000-TF containing different ratios of each substance. In addition, the lipid components of HSPC/Chol/DSPE-PEG2000/TP (69:9:12:12, mass ratio) and HSPC/Chol/DSPE-PEG2000/TP (58:14:4:22, molar ratio) were separately used for several steps, including the preparation of blank and TP liposomes (TP@LIP). To obtain a thin film, HSPC, Chol, DSPE-PEG2000, and TP were dissolved in absolute ethanol in a pear-shaped bottle before drying on a rotary evaporator at 37 °C. An appropriate amount of isotonic buffer was then added, mixed well, and hydrated at atmospheric pressure for 30 min before ultrasonication (SM-1000D, SHUNMATECH, Co., Ltd. Shanghai, China). The resulting mixture was then passed through 0.2 μm and 0.1 μm polycarbonate fibro-lipid membranes six times to obtain the liposome solution. The TF-modified tretinoin liposomes (TF-TP@LIPs) were obtained by incubating the above-mentioned tretinoin liposomes with an appropriate amount of synthetically resoluble TF-PEG2000-DSPE lyophilized powder solution at 60 °C for 1 h. Liposomes loaded with Coumarin 6 (TF-C6@LIP, C6@LIP) were also prepared using the above procedure. To evaluate the stability of the TF-TP@LIP and TP@LIP, the samples were placed in a penicillin bottle and stored at 4 °C. The particle size and PDI (polydispersity index) were determined after 0, 1, 2, 3, 4, 5, 6, and 7 days.

### 2.5. Characterization of Liposomes

Dynamic light scattering with a Zetasizer Nano ZS90 (Malvern, UK) was used to determine the particle size, polydispersity index, and zeta potential for all liposomes, while transmission electron microscopy (TEM) was used to observe their morphologies (HT7800, Hitachi, Tokyo, Japan). Furthermore, high-performance liquid chromatography (HPLC) was used for measuring the TP content (Alliance HPLC E2695, Waters Corporation, Milford, MA, USA). The unencapsulated drug was separated from the liposome solution by centrifugal ultrafiltration (4500 rpm, 5 min) (TG16-WS, Changsha, China), and the encapsulation rate was determined by HPLC. Specifically, the method involved the addition of 20 volumes of methanol to the liposome solution, followed by demulsification using ultrasound for 20 min and measurement of the total TP amount (Wtotal). In addition, 0.2 mL liposome solution was measured precisely into an ultrafiltration centrifuge tube (MWCO 30,000 Da), diluted to 1 mL with PBS, and centrifuged (4500 rpm, 5 min) to obtain the unwrapped TP. The unwrapped drug content (W_Unwrapped drug_) was determined, and the encapsulation efficiency (EE) was calculated as:

EE% = 1 − (W_Unwrapped drug_/W_total_) × 100%,

where W_Unwrapped_ and W_total_ represent the unencapsulated amount and the total amount of the drug, respectively. The drug loading (DL%) for TP-loaded liposomes was then calculated using the following equation:

DL% = (WTP − W_Unwrapped drug_/W_Total amount of lipids_) × 100%.

### 2.6. In Vitro Drug Release from LIPs

The drug-releasing properties of TP@Lip and TF-TP@Lip were evaluated in vitro using dialysis, as previously reported [33]. For this purpose, equal volumes of TP@Lip, TF-TP@Lip, and TP solution (5 mL) were placed in dialysis bags (MWCO 8–14 kDa), which were then placed in 200 mL of PBS (pH 7.2~7.4) containing 1% Tween 80. The bags were gently shaken at 100 rpm (37 °C), and at predetermined intervals (0.25, 0.5, 1, 2, 4, 6, 8, 10, and 12 h), 2 mL of the surrounding buffer was collected and replaced with fresh buffer. The samples were analyzed by HPLC to determine the TP content. The cumulative release rate was then calculated and plotted for each time point (*n* = 3).

### 2.7. Hemolytic Examination of TF-TP@LIP

One milliliter of mouse blood was collected into an anticoagulation tube and centrifuged. After removal of the plasma layer, an appropriate amount of physiological saline was added to the tube and mixed. This was followed by centrifugation at 2000 rpm for 5 min, and after removing the supernatant, saline was again added. The above procedure was repeated two to three times, with the resulting erythrocytes made up to a 2% (*v*/*v*) suspension with saline and set aside. Corresponding solutions were subsequently added in the order shown in Table 1 with proper mixing prior to a 2 h incubation at 37 °C. Hemolysis and coagulation reactions were observed for each tube (tubes No. 1 to 3 represented different concentrations of the test samples, No. 4 was the negative control, and No. 5 was the positive control). Finally, after the incubation, the tubes were centrifuged (500 rpm, 5 min), with the resulting supernatant collected to determine the absorbance (OD) of each well at 540 nm. Based on the results, the hemolysis rate was determined according to the following equation:

Hemolysis rate (%) = OD_Samples_OD_Negative_/OD_Positive_ − OD_Negative_

### 2.8. Cellular Uptake

Fluorescent-labeled Coumarin 6 liposomes (C6@LIP) and TF-modified Coumarin 6 liposomes (TF-C6@LIP) with different coupling densities were investigated by flow cytometry of HepG2 cells. The cells were cultured (5 × 10^5^ cells/well) in 12-well plates for 24 h to allow attachment. C6@LIP and TF-C6@LIP, with different coupling densities, were added to the cultures, with blank liposomes acting as blank controls. The final concentration of Coumarin 6, in this case, was 50 ng/mL. Cells were collected when in the logarithmic growth phase and treated with TF-modified Coumarin-6 liposomes before incubation for 6 h. The cells were then washed with PBS, trypsinized, and centrifuged at 1000 rpm for 5 min. The cells were resuspended in PBS and the fluorescence intensities measured using flow cytometry (Gallios, Beckman Coulter, Brea, CA, USA).

Fluorescence microscopy was also used to verify the uptake of C6@LIP or TT-C6@LIP by HepG2 cells. C6, at a final concentration of 50 ng/mL, was incubated with HepG2 cells together with C6@LIP and TF-C6-LIP, with blank liposomes as a blank control. After incubation for 6 h, the cells were washed with PBS, fixed with 4% paraformaldehyde for 15 min at room temperature (RT), and stained with DAPI. A fluorescence microscope (Axio Vert A1, Zeiss, Germany) was then used for imaging the HepG2 cells.

To verify whether HepG2 cells could take up TF-TP@LIP through TFR-mediated endocytosis, a 20-fold excess of free TF was preincubated with the cells before the competitive inhibition experiment.

### 2.9. In Vitro Cytotoxicity

To evaluate the cytotoxicity of TF-TP@LIP in vitro, different concentrations of TF-TP@LIP were added to cells in 96-well flat-bottomed plates (8 × 10^3^ cells/well) and grown overnight. After the addition of 5 mg/mL of MTT, the cells were incubated for a further 4 h, after which the culture supernatants were discarded, and 100 μL of DMSO was added. Absorbances were measured at 490 nm with an EnVision Multimode Plate Reader (PerkinElmer, Waltham, MA, USA) to assess cell viability. Comparative studies were also conducted using TP@LIP and the free drug [34].

### 2.10. Analysis of Apoptosis and the Cell Cycle by Flow Cytometry

To observe cellular apoptosis, HepG2 cells were first incubated with TF-TP@LIP, TP@LIP, and TP alone (TP was administered at a final concentration of 80 nM) for 24 h. The cells were then washed twice with cold PBS, and after being collected using EDTA-free trypsin, were resuspended in binding buffer and stained with Annexin-V-FITC and PI solution (BD, China) for 30 min at room temperature, before analysis by flow cytometry to assess apoptosis.

HepG2 cells were cultured in 6-well plates at a density of 3 × 10^5^ cells/well for 24 h. TP, TP@LIP, and TF-TP@LIP (TP was administered at a final concentration of 80 nM) were then added, and after another 24 h incubation, the cells were resuspended in 1 mL of RNase A solution, according to the instructions of the cell cycle assay kit (MULTI SCIENCE, Hangzhou, China). This was followed by incubation for 30 min in a water bath at 37 °C before measurement of the cell cycle distribution by flow cytometry.

### 2.11. Animal Tumor Models

Based on previous methods, BALB/c nude mice were used to establish in vivo tumor models. The mice were injected with a 200 μL suspension of HepG2 cells (approximately 6×10^6^ cells suspended in PBS) on each side (near the hind limb) prior to fluorescence imaging of the tumors. The growth of the tumors was monitored, and the volumes were calculated as follows:

tumor volume (mm^3^) = A × B2/2,

where “A” and “B” represent the longest and shortest diameters of the tumor, respectively.

### 2.12. Live Fluorescence Imaging

Nude mice were injected with TF-DiR@LIP, DiR@LIP, and DiR via the tail vein at a dose of 0.1 mg/kg (*n* = 3). After 1, 2, 4, 6, 8, 12, and 24 h, the animals were anesthetized by isoflurane inhalation, and the distribution of the nano-formulation in vivo was visualized using an IVIS Spectrum Imaging System (Axio Vert A1, Zeiss, Germany). After 24 h, the mice were euthanized, and tissues (heart, liver, spleen, lung, kidney, and tumor tissues) were harvested, dissected, and imaged.

### 2.13. Safety Evaluation

At the end of treatment, blood was collected from the mice, centrifuged to obtain the serum, and used to measure the liver index (ALT/AST/TP/ALB) and kidney index (BUN/CRE/UA). Heart, liver, spleen, lung, and kidney tissues were also dissected, and after being weighed, were fixed in paraformaldehyde, paraffin embedded, and cut into 30 μm thick sections. The sections were then stained with hematoxylin–eosin (H&E) and evaluated for morphological changes, signs of inflammation, necrosis, or cell damage.

### 2.14. Statistical Analysis

All experimental data are presented as the mean ± SD. Statistical analysis was performed using a one-way ANOVA for multiple samples, as well as a Student’s *t*-test for paired sample sets. For all analyses, *p*-values less than 0.05 were considered statistically significant, while those less than 0.01 were considered extremely significant. For all analyses, GraphPadPrism 6.0 software was used.

## 3. Results and Discussion

### 3.1. Characterization of the Liposomes

**Figure 1:**
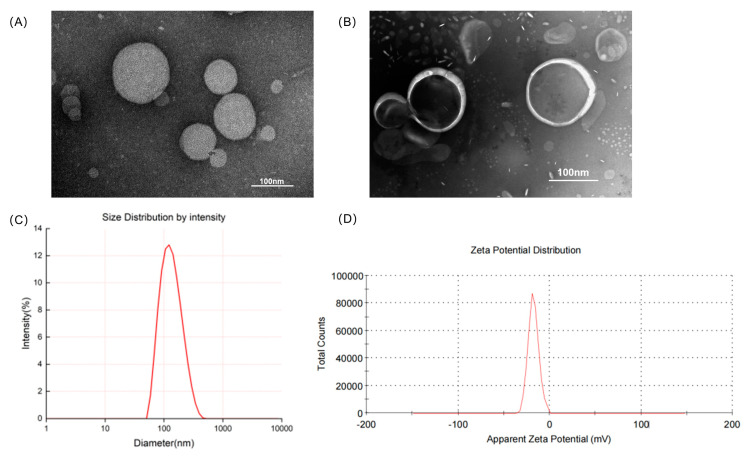
Characteristics of the liposomes. (**A**) Transmission electron microscopy images of TP@LIP; (**B**) Transmission electron microscopy images of TF-TP@LIP; (**C**) Particle size distribution of TF-TP@LIP; (**D**) Zeta potentials of TF-TP@LIP.

The physical properties of liposomes normally affect the amount of drugs that can be loaded, as well as their injectability, biodistribution, in vivo clearance, shelf life, and dose prediction and the pharmacokinetic profile of the drug administered [35]. In this study, thiolated TF was allowed to react with DSPE-PEG2000-MAL to yield DSPE-PEG2000-TF, a functional phospholipid with targeting potential. A thin-film dispersion method was also applied to prepare TF-TP@LIP, and a diluted solution of the latter was observed under TEM. In this case, results showed that TF-TP@LIP was spherical and uniformly distributed (Figure 1). Table 2 provides further information on the physical properties of the liposomes, including the particle size, polydispersity index (PDI), zeta potential, encapsulation efficiency, and drug loading capacity.

Based on the USP pharmacopeial forum (USP-PF), the mean globular diameter of particles for IV administration should be less than 500 nm, as determined by DLS, irrespective of the lipid concentration [36]. TP@LIP was found to have a mean particle size of 121.10 ± 3.40 nm, a polydispersity index of 0.15 ± 0.03, and a zeta potential of −17.50 ± 0.20 mV, while the values for TF-TP@LIP were 130.33 ± 1.89 nm, 0.20 ± 0.04, and −23.20 ± 0.90 mV, respectively. Therefore, if the particle size of a long-circulating liposome is less than 150 nm, it would be able to penetrate the blood vessels in the tumor area effectively through the enhanced permeability and retention effect (EPR), leading to enrichment within the tumor region. At the same time, this would improve the distribution of the drug in the body, while reducing its toxicity. Interestingly, the particle size of the liposomes prepared in this study was about 130 nm, which is conducive to their passive targeting to reach the tumor tissue [37]. Incubation of TP@LIP with TF-PEG2000-DSPE resulted in an increase in particle size with no significant change in the PDI, indicating that the LIP had a homogeneous particle distribution and that its reproducibility, obtained by the thin-film dispersion method, was high (Figure 1 and Table 2).

The encapsulation rates (EE%) for the TP@LIP and TF-TP@LIP liposomes were 88.23 ± 0.14% and 85.33 ± 0.41%, respectively (Table 2). In our previous study, we investigated three methods for determining the encapsulation of fat-soluble drugs by liposomes, namely, low-speed centrifugation, dextran gel columns, and ultrafiltration centrifugation. The ultrafiltration centrifugation method does not require much equipment and saves time. This method can also assess drug encapsulation more accurately, and thus the ultrafiltration centrifugation method was selected for determining the encapsulation rate of triptolide liposomes (Supplementary Section S2). Since the ultrafiltration tube can adsorb some of the drug, pre-saturation treatment is required before the ultrafiltration centrifugation operation. In addition, due to the “concentration polarization” effect, the membrane permeability of free drugs tends to be low, so the liposome solution to be tested should be diluted during the experiment to avoid this phenomenon [32].

These results indicated that TF-TP@LIP had a uniform particle size distribution and good electronegative properties. It was also within the nanoscale size and had good physicochemical characteristics, which could potentially regulate the pharmacokinetic and pharmacodynamic characteristics of drugs, thereby improving their therapeutic indices [38].

**Figure 2:**
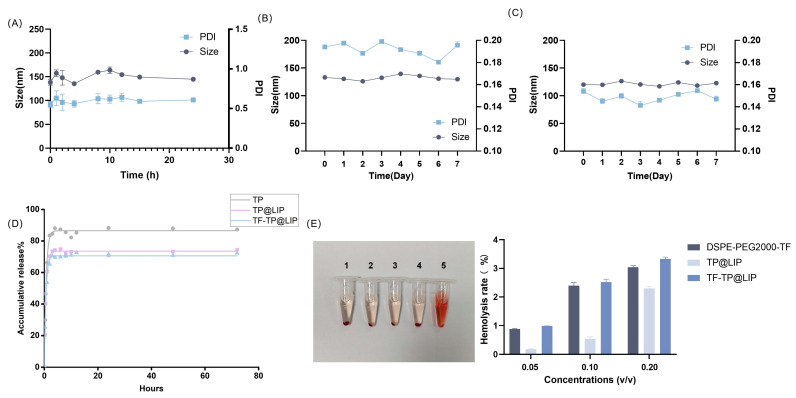
Liposome stability, drug release, and induction of hemolysis. (**A**) Serum stability of TF- TP@LIP; (**B**) Stability of TF-TP@LIP; (**C**) Stability of TP@LIP; (**D**) Release profiles of TP, TP@LIP, TF-TP@LIP; (**E**) Induction of hemolysis. (1. DSPE-PEG2000-TF; 2. TP@LIP; 3. TF-TP@LIP; 4. Negative control group; 5. Positive control group).

To investigate the serum stability of TF-TP@LIP, 10% FBS was prepared as the medium, and the particle size distribution, as well as PDI of TF-TP@LIP, were quantified with DLS. It was found that the single peak of the particle size distribution of TF-modified ryanodine liposome was still present after mixing with 10% fetal bovine serum. As shown in Figure 2A, the particle size distribution and PDI of TF-TP@LIP did not change significantly within 24 h after mixing with the serum, thereby indicating good serum stability for TF-TP@LIP. In addition, the results of the stability experiment showed that the particle size did not increase after 7 days in TP@LIP and TF-TP@LIP. This demonstrates good stability for TP@LIP and TF-TP@LIP (Figure 2B,C).

### 3.2. In Vitro Release of TF-TP@LIP

The in vitro release of free TP, TP@LIP, and TF-TP@LIP was investigated using dialysis, and the results are shown in Figure 2D. Due to the rapid release of the TP on the surface of liposomes, there was an initial release of the drug, but this changed to a slow-release phase after 1 h. To gain insight into the release mechanism, three mathematical models, including the first-order, Korsmeyer–Peppas, and Weibull models, were utilized to predict the release behavior of TP; the results are shown in the in Supplementary Materials (Section S5). From the kinetic parameters (Section S5), it appears that the experimental data are best described by the first-order model (Figure 2D). The release experiments further showed that both TP@LIP and TF-TP@Lip had similar drug-release behavior, indicating that the combination with TF had no significant effects on the release behavior of the TP liposomes. The slow-release characteristic at this phase could be attributed to the fact that TP was held by the lipid, and therefore, TP was released gradually from the lipid matrices mainly through dissolution and diffusion. These results showed that TP could be released from the two lipids. Such slow release in the body is not only beneficial to reducing leakage of the drug in the blood circulation before reaching the tumor tissue, but also effectively enhances the stability of the drug.

### 3.3. Hemolytic Activity of Liposomes

Liposomes are commonly administered intravenously. However, many factors within the bloodstream can disrupt liposomes. For example, phospholipases in the circulatory system hydrolyze phospholipids, high-density lipoproteins disrupt phospholipid membranes, and various modulators of the complement system, such as antibodies and complement, bind to liposomes, thus disrupting the hydrophilic channels in the phospholipid membrane. This leads to leakage of the encapsulated drugs and the entry of water and electrolytes, while accelerating liposome clearance. Therefore, in this study, liposome stability was evaluated by examining liposome–serum interactions [30]. The synthesis of functional phospholipids (DSPE-PEG2000-TF) involves various reactions and reaction materials. To investigate the biosafety of this synthetic material, the hemolysis rate was quantified using a multimode plate reader. It was clear, from the results of the quantitative analysis, that the hemolysis rate was less than 5%, indicating that the synthesis and preparation were safe. The results are shown in Figure 2E.

### 3.4. Cellular Uptake and Targeting Effects In Vitro

**Figure 3:**
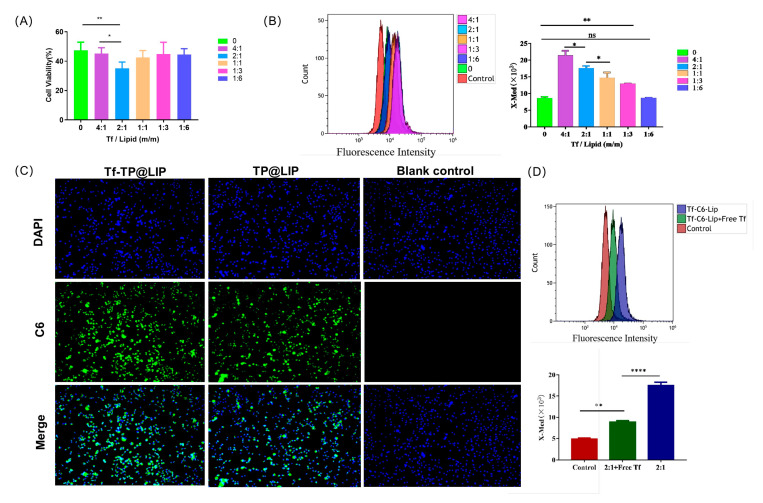
Uptake of liposomes by cells. (**A**) Anti−tumor activity of TF−TP@LIP with different coupling densities; Uptake of TF−TP@LIP investigated by flow cytometry (**B**) and fluorescence microscopy (**C**) (scale bar: 100 µm). (**D**) Uptake of TF−TP@LIP by HepG2 cells. (**** *p* < 0.0001, ** *p* < 0.01, * *p* ≤ 0.05, ns, *p* > 0.05).

The results of the MTT assay showed that the anti-tumor activity of TF-TP@LIP was dependent on the TF coupling density. When the TF/lipid ratio was 2:1, TF-TP@LIP showed the greatest inhibitory effect on cells (Figure 3A). This may have been due to the high affinity of TF-TP@LIP in binding to TFR to reach saturation. Based on existing studies describing the mechanism of TP endocytosis, C6 dye was used instead of TP to determine the intracellular uptake and distribution of liposomal vectors [39]. TFR was strongly expressed on the surfaces of the human hepatoma HepG2 cells. These cells were used to assess the in vitro targeting of TF-TP@LIP. Targeted fluorescent liposomes (TF-C6@LIP) and non-targeted fluorescent liposomes (C6@LIP) with different coupling densities were also incubated with HepG2 cells for 4 h. The flow cytometry results (Figure 3B) showed that the fluorescence intensity of the targeted liposomes in the cells was significantly higher than that of non-targeted liposomes, thus confirming the fluorescence microscopy findings (Figure 3C). The observations also suggested that the TF modification enhanced the uptake of liposomes by HepG2 cells. In addition, the uptake of TF-C6@LIP by the cells increased with the degree of TF modification (Figure 3B).

To verify whether HepG2 cells take up TF-TP@LIP through TFR-mediated endocytosis, a 20-fold excess of free TF was preincubated with the cells in a competitive inhibition experiment. The results (Figure 3D) of this experiment indicated that uptake of TF-C6@LIP by the HepG2 cells was significantly decreased, thereby suggesting that TF-C6@LIP targeting of the TFR enhanced liposome targeting of the cells. Studies [30,40,41] have confirmed that TF-modified liposomes can even enter tumor cells through receptor-mediated endocytosis, indicating that the targeting by TF-TP@LIP could be related to TFR-mediated endocytosis. This would lead to larger amounts of TP entering the tumor tissue due to the strong affinity of the receptor-mediated interaction and its ability to transport the TF-modified nano-targeted liposomes into hepatoma cells.

### 3.5. Anti-tumor Effects of TF-TP@LIP In Vitro

**Figure 4:**
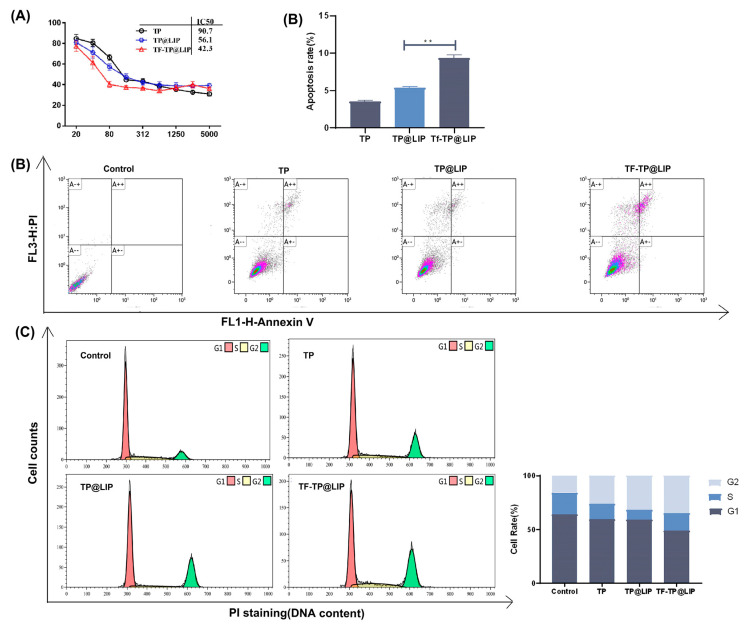
The effects of TF-TP@LIP on the proliferation, apoptosis, and cell cycle of HepG2 cells. (**A**) Cytotoxicity of TP, TP@LIP, and TF-TP@LIP in HepG2 cells after 48 h; (**B**) Flow cytometric analysis and quantification of apoptosis in HepG2 cells following treatment with Control, Free TP, TP@LIP, and TF-TP@LIP for 24 h at an equivalent TP concentration; (**C**) Flow cytometric analysis and quantification of cell cycle distribution of HepG2 cells following treatment with Control, free TP, TP@LIP, and TF-TP@LIP for 24 h at equivalent TP concentrations. (** *p* < 0.01).

To investigate the inhibitory effects of TP@LIP on hepatocellular carcinoma cells, TP, TP@LIP, and TF-TP@LIP groups with different mass concentrations were set up to compare the effects of drug administration. As shown in Figure 4A, the IC_50_ values of HepG2 cells in the free TP, TP@LIP, and TF-TP@LIP groups were 90.6 nM, 56.1 nM, and 42.3 nM, respectively. The results further demonstrated that the anti-tumor effects of TP were effectively improved after encapsulation with TF-modified liposomes, and indicated that TF did not undergo structural changes and lose its biological function by the modification, which was the basis for TF-TP@LIP to target tumor tissues. Notably, MTT assays showed that inhibition of HepG2 cell proliferation by TF-TP@LIP was affected by the density of the TF modification. When the TF-to-lipid mass ratio was 2:1, TF-TP@LIP LIP significantly reduced cell viability compared to TP, TP@LIP, and other densities of TF-TP@LIP.

Flow cytometry showed that TF-TP@LIP significantly increased the percentage of apoptotic cells in comparison with the free TP and TP@LIP groups (** *p* < 0.01) (Figure 4B). In addition, a comparison of the distribution of cells in the phases of the cell cycle (G0/G1, S, and G2-M phases) among the different treatment groups indicated that treatment with TF-TP@LIP increased the percentage of cells in the G2-M phase and significantly decreased those in the G1 phase, compared with the free TP and TP@LIP groups. This indicated that TF-TP@LIP increased cell cycle arrest and thus inhibited hepatocellular carcinoma cell HepG2 proliferation (Figure 4C). These results confirmed that TF was stably bound to the surface of liposomes and effectively enhanced their uptake by TFR-expressing cancer cells. Studies [42,43] have shown that if DNA damage cannot be repaired during the cell cycle, cells will initiate an apoptotic program, causing the death of cells with DNA damage, as suggested here. Compared with the control group, both the TF-TP@LIP groups showed significantly enhanced cycle arrest and promotion of apoptosis, which could be related to the fact that TF modification enhanced the uptake of the liposomes by hepatoma cells.

### 3.6. In Vivo Real-Time Imaging

**Figure 5:**
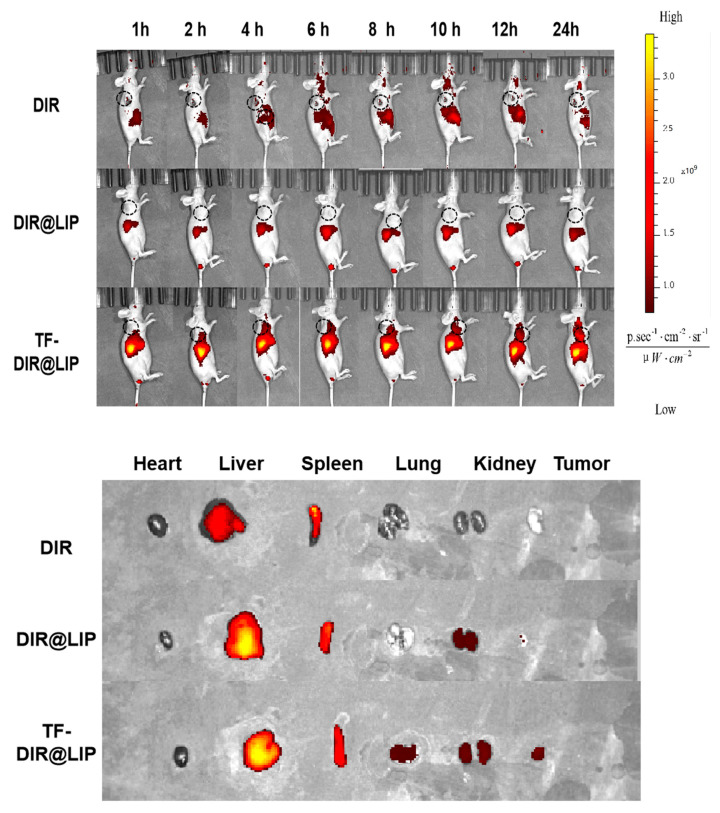
Results of in vivo imaging after tail vein injection of DIR, DIR@LIP, and TF-DIR@LIP in tumor-bearing nude mice. Tissue distribution 24 h after injection in the different treatment groups.

In vivo imaging enables non-invasive real-time monitoring and quantitative analysis of tumor growth and metastasis in living animals. It can also be used to observe preparations targeting tumor organs through in vivo imaging [43]. The present study observed in vivo NIRF imaging of TF-DiR@Lip in tumor-bearing mice to determine its bio-distribution. In this study, DiR, a fluorescence probe, was loaded into liposomes to mimic TP. The intensity of the fluorescence signals represents the accumulation of the drug in the mice. As shown in Figure 5, there was rapid accumulation of the DIR-labeled TF-DIR@LIP over time, with higher fluorescence intensity visible in the tumor tissues 4 h after injection. However, no obvious fluorescence accumulation was seen in the tumor tissues of the DIR-labeled DIR@LIP (non-targeting control) and free DIR mouse groups. Tumor tissue fluorescence was only seen in the DIR@LIP group, demonstrating that TF-TP@LIP was effective for tumor targeting.

### 3.7. In Vivo Anti-Hepatoma Efficacy

**Figure 6:**
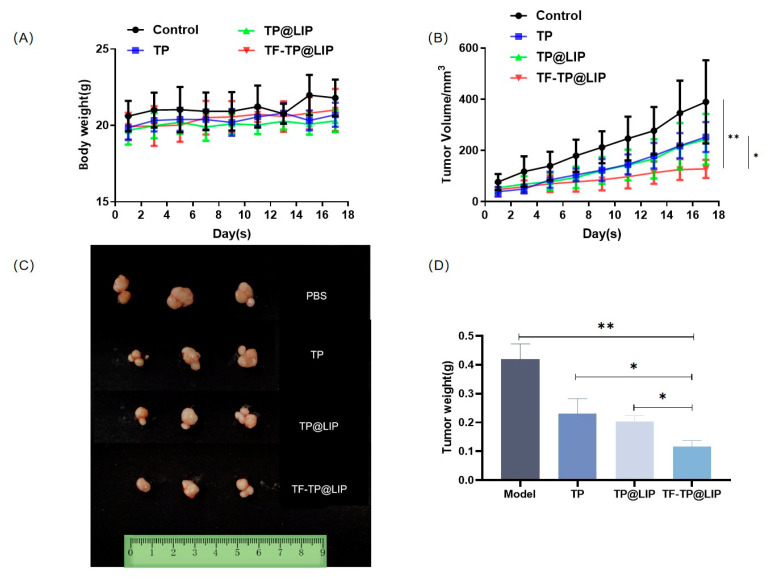
Growth inhibition of HepG2-cell xenograft tumors in the different treatment groups. (**A**) Body weight; (**B**) Tumor volume; (**C**,**D**) Morphology tumor tissue after treatment in different treatment groups (** *p* < 0.01, * *p* < 0.05).

**Figure 7:**
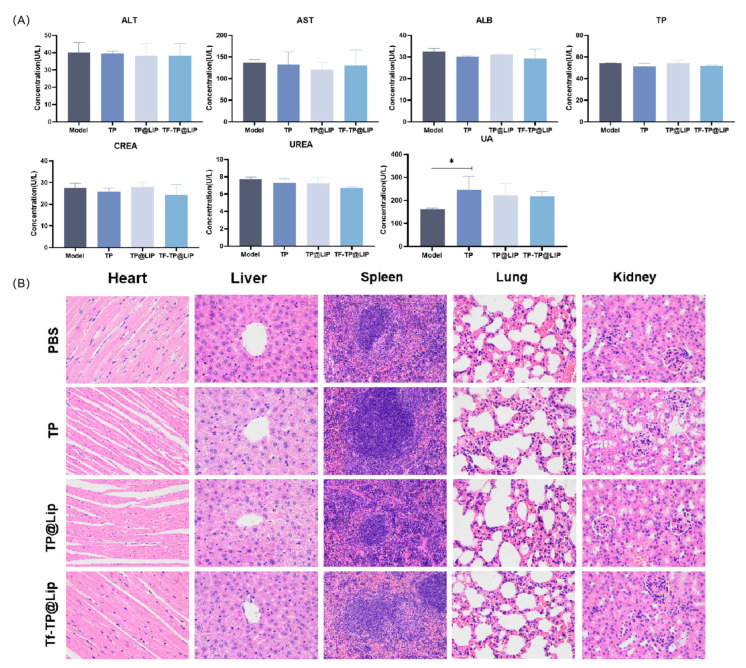
Biochemical indices and histological sections. (**A**) Blood biochemical tests; (**B**) H&E-stained sections of heart, liver, spleen, lung, and kidney tissues from mice in the different treatment groups (400×). (* *p* < 0.05).

When the tumor volumes reached 120–150 mm^3^, the mice were randomly divided into four groups according to the principle of even distribution of tumor size, namely, the control, TP, TP@LIP, and TF-TP@LIP groups. The drug was administered through the tail vein at a dose of 0.2 mg/kg TP in a volume of 200 μL. A total of seven doses was administered. Tumor suppression was monitored for 18 days, and the body weights, as well as tumor volumes, in each group were measured and recorded every other day from the first day of administration. Compared with other groups, there was significant inhibition of tumor growth in the TF-TP@ LIP group. At the end of the treatment, the tumor volumes and tumor weights of the TF-TP@ LIP group were visibly smaller than those of the other groups (Figure 6). After drug administration, the main organs of nude mice, namely, the heart, liver, spleen, lung, and kidney, were collected for evaluation using H&E staining of tissue sections. The morphological changes of the organ tissues of each group are shown in Figure 7. The organ tissues of the free TP group showed obvious changes, such as the infiltration of inflammatory cells and disordered tissue, indicating toxicity of the free drug. Compared with the control group, no abnormal changes were found in the major organs of the mice in the TF-TP@ LIP treatment group. In addition, the values of the main liver and kidney parameters in the TF-TP@ LIP group were within the normal range and did not differ significantly from those in the control group, demonstrating the safety of the formulation.

## 4. Conclusions

Drug delivery systems using transferrin-mediated liposome targeting show promise for cancer therapy. In this study, a TF-modified liposome, targeting the TFR on the surface of hepatocellular carcinoma cells, was designed for the delivery of TP. This active targeting delivery system had good biocompatibility and low toxicity. Compared with free TP and non-targeted TP liposomes, the addition of TF significantly enhanced hepatoma cell cytotoxicity, as well as showed enhanced uptake of the TF-TP@LIP in vitro. The inclusion of TF also increased cell cycle arrest and promoted apoptosis in hepatoma cells. The results of in vivo experiments further showed that TF-TP@LIP had stronger anti-tumor effects in vivo compared with the free TP TP@LIP groups, and the results of in vivo and in vitro studies were consistent. In addition, the toxicity of TF-TP@LIP was evaluated by changes in tissue morphology, observed by H&E staining of pathological sections. Compared with the control group, pathological changes were observed in sections of heart, liver, lung, and kidney tissues in the TP group due to toxicity and side effects, while the tissue structures in the TF-TP@LIP group were normal, indicating the relative safety of the preparation and that the toxicity of TF-TP@LIP was less than that of free TP. Therefore, the use of transferrin for targeting TP-containing liposomes may prove to be an effective nanomedicine for the treatment of liver cancers.

## Figures and Tables

**Table 1:** Hemolysis experiment design table.

Test Tube	1	2	3	4	5
2% Red Blood Cells Suspension (μL)	500	500	500	500	500
Saline solution (μL)	450	400	300	500	
Distilled water (μL)					500
Sample (μL)	50	100	200		

**Table 2:** Physicochemical properties of liposomes. Particle size, zeta potential, encapsulation efficiency, and drug loading of liposomes (unmodified and modified with transferrin).

Liposomes	Size (nm)	PDI	Zeta Potential (mV)	EE%	DL%
TF-Blank@LIP	125.02 ± 2.20	0.19 ± 0.05	−23.90 ± 2.30		
TP@LIP	121.10 ± 3.40	0.15 ± 0.03	−17.50 ± 0.20	88.23 ± 0.14	10.26 ± 0.02
TF-TP@LIP	130.33 ± 1.89	0.20 ± 0.04	−23.20 ± 0.90	85.33 ± 0.41	9.96 ± 0.21

## Data Availability

The data are not publicly available due to the contents of the graduation thesis are subject to confidentiality.

## Supplementary Materials

The following supporting information can be downloaded at: https://www.mdpi.com/article/10.3390/biomedicines11102869/s1, Figure S1: Size histogram of TEM images of TP@LIP; Figure S2: Size histogram of TEM images of TF-TP@LIP; Figure S3: Elution Curve of TP(A) and TP@LIP(B); Table S1: The choice of centrifugal strength; Table S2: The choice of centrifugal time; Table S3: Ultrafiltration recovery rate of free TP.
