# Supplementary material for: Transferrin-Modified Triptolide Liposome Targeting Enhances Anti-Hepatocellular Carcinoma Effects
Source: Biomedicines. 2023 Oct 23;11(10):2869. doi: 10.3390/biomedicines11102869 (PMC10604558; doi:10.3390/biomedicines11102869)
Supplement: Supplementary file 1 [file biomedicines-11-02869-s001.zip › biomedicines-2615489-supplementary.pdf]

S1. Size histogram of TEM images of TP@LIP and TF-TP@LIP

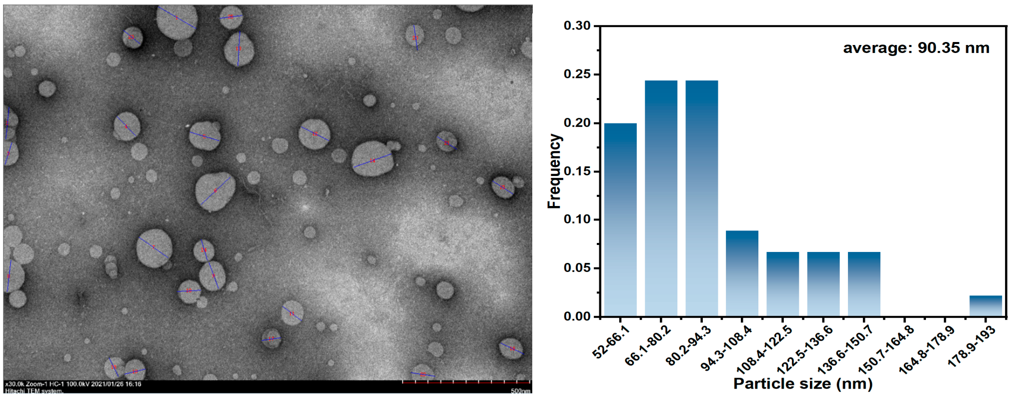

Figure S1 Size histogram of TEM images of TP@LIP

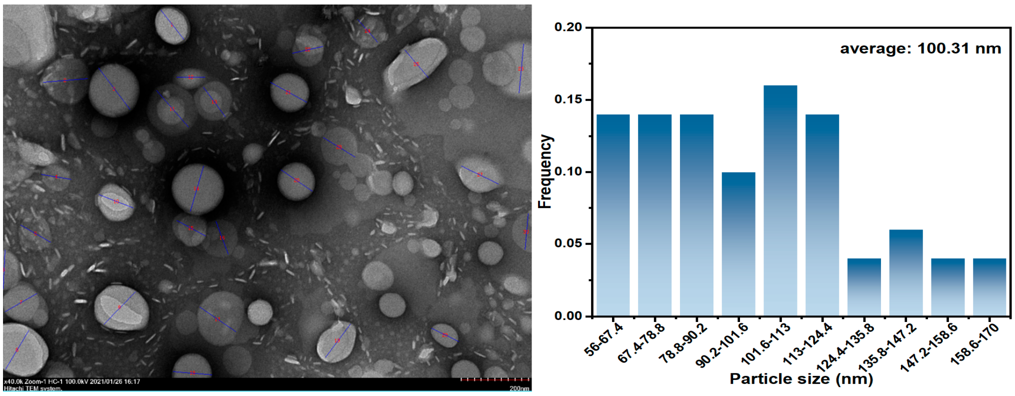

Figure S2. Size histogram of TEM images of TF-TP@LIP

S2 Three methods for determining the encapsulation efficiency of liposomes

S2.1 Dextran gel column method

S2.1.1 Treatment of dextran gel column

Firstly, a proper amount of G-50 glucan gel was weighed and placed in a beaker, and a certain volume of PBS solution was added to swell for more than 2h. Glucan gel G-50, which was swollen and mixed with PBS, was added to the chromatographic column through glass rod drainage, and bubbles were expelled by tapping the outer wall of the chromatographic column constantly to keep the water flow at the outlet smooth, and PBS was added in time to prevent the column from drying out. The gel after loading the column must be uniform without bubbles or obvious stripes. Before loading, the gel column was balanced with PBS buffer for about 30 minutes to stabilize the

baseline.

S2.1.2 Sample loading, elution, and elution curve drawing

The methanol solution of triptolide was accurately measured and diluted to 400 μL with deionized water, then column chromatography was performed on the upper column and eluted with ultra-pure water at the flow rate of 1mL/min. The flow fraction was collected in stages, 1 tube was collected every 1 mL, and a total of 40 tubes were collected. After centrifugation and concentration of each tube, the samples were dissolved with methanol and demulsified by ultrasound and then the peak area was determined under the chromatographic conditions of item. The content of triptolide was determined and the recovery rate of the free drug was calculated.

Accurately measure 400 μL TP@LIP, place the sample on the column, and separate the collected solution by the same method described in 2.1.2. The collected solution is treated and tested in the same way, and the elution curve is drawn according to the peak area value and the number of tubes.

S2.2. Low-speed centrifugal method

The prepared triptolide liposome was transferred to a 5 mL volumeable bottle with PBS and mixed evenly. 1mL liposome solution of triptolide was precisely absorbed, followed by ultrasonic demulsification with the appropriate amount of methanol, then filtered by 0.22 μm filter membrane, 20 μL was taken into the sample for analysis, and the total dosage(W_{total})was calculated. Another 1mL liposome solution was taken and placed in a centrifuge tube, centrifuged at 2500rpm for 8min. The supernatant was discarded and a 1mL methanol water bath was added for ultrasonic dissolution and precipitation. After filtration with a 0.22 μm filter membrane, the drug content ($W_{Unwrapped\ drug}$)that was not encapsulated in the liposome was calculated.

S2.3. Ultrafiltration centrifugal method

An appropriate amount of liposome solution was precisely measured, 20 times the amount of methanol was added, and the total amount of triptolide (W_{Total}) was determined by ultrasonography for 20 min. In addition, 0.2 mL liposome solution was precisely measured in an ultrafiltration centrifuge tube (MWCO 30,000Da), diluted to

1 mL with PBS, and centrifuged (4500 rpm, 5 min) to obtain free triptolide. The free drug content ($W_{Unwrapped\ drug}$) was determined, and the encapsulation rate was calculated.

$EE\% = 1-(W_{Unwrapped\ drug}/W_{Total}) \times 100\%$

S3.Results

S3.1 Elution curve of liposomes by dextran gel column method

The glucan gel column method uses the principle of a molecular sieve to separate the liposome with a large molecular weight difference from the free drug, and the liposome with a large molecular weight is eluted first, and the free drug is eluted. The solution was collected in different discharge periods, and the peak area was measured by HPLC. The elution curve was drawn. As can be seen from Figure 3, both the free drug and the liposome solutions were collected in 10 to 25 tubes, preventing the effective separation of the liposome from the free drug.

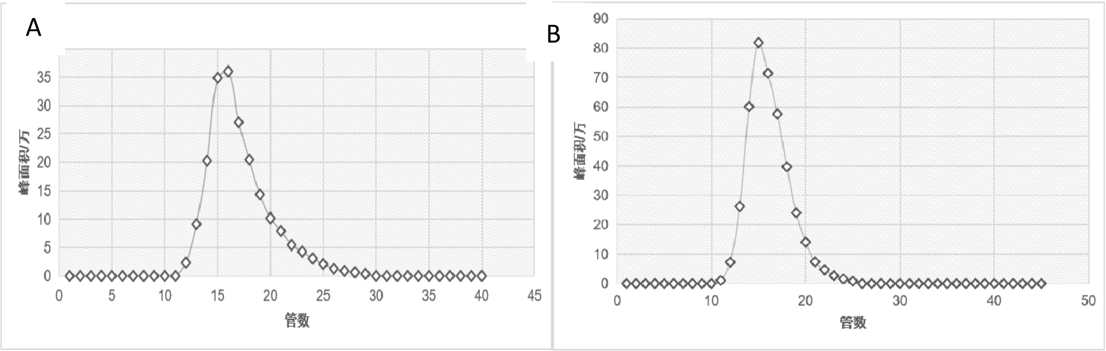

Figure S3 Elution Curve of TP(A) and TP@LIP(B)

S3.2 Results of entrapment efficiency by low-speed centrifugation

S3.2.1 Selection of the centrifugal rotation speed

The fixed centrifugation time was unchanged at 4 min, and the resolution of the liposome and free TP at different speeds was investigated. The results showed that when the centrifugal speed reached 2500 rpm, the resolution was maintained at about 46%, and the increasing speed had almost no effect on the resolution, so the low-speed centrifugal speed was 2500 rpm. The results are shown in Table 1.

Table S1. The choice of centrifugal strength

Centrifugation speed	1000	1500	2000	2500	3000

（rpm）					
Average peak area （n=3）	1647064	1746681	1885253	1960711	1913564
Drug content (mg)	0.040	0.043	0.046	0.048	0.047
resolution （%）	39.12	41.61	44.94	46.71	45.63

S3.2.2 Selection of the centrifugation time

The fixed centrifugation speed was unchanged at 2500 rpm, and the separation of liposomes and free TP was investigated after different centrifugations. The experimental results showed that at the centrifugation speed of 2500 rpm, the centrifugation time reached 8 min, and the resolution of the two was almost unchanged, so 8 min was chosen as the centrifugation time, and the results are shown in Table 2.

Table S2. The choice of centrifugal time

Centrifugation time (min)	2	4	6	8	10
Average peak area（n=3）	2026695	2022245	2082548	2317961	2270566
Drug content (mg)	0.049	0.049	0.051	0.057	0.055
Resolution （%）	47.21	47.84	48.82	55.23	53.91

S3.2.3 Entrapment efficiency measurement results

According to 2.2. Low speed centrifugation method, the liposomes and free drugs were separated, and the average entrapment efficiency of triptolide liposomes was measured (74.2±3.18) %.

S3.3. Results of entrapment efficiency by ultrafiltration centrifugation

S3.3.1 Recovery rate of free drugs

According to the recovery rate and the peak area of the drug without ultrafiltration treatment, the average recovery rate of free TP was 98.6% and the RSD value was 3.5% (n=4), indicating that the ultrafiltration membrane has no adsorption effect on the drug. The results are shown in Table 3.

Table S3. Ultrafiltration recovery rate of free TP

No.	Free drug recovery rate（%）	Mean value（%）	RSD（%）
1	97.64	98.63	3.53

2	94.21
3	102.13
4	100.54

S3.3.2 Entrapment efficiency determination results

Three batches of triptolide liposomes were prepared in parallel, and the encapsulation efficiency of TP@LIP was determined by ultrafiltration centrifugation method. The results show that the average encapsulation rate of TP@LIP is (53.6±1.41) %.

S4. Discussion

This section examines three methods suitable for determining entrapment efficiency, including low-speed centrifugation, glucan gel column, and ultrafiltration centrifugation. The low-speed centrifugation method is under the action of centrifugal force, according to the specific gravity of the liposome and the free drug, to achieve the separation of the two. Glucan gel column method has the dual effect of molecular sieve and adsorption to achieve the purpose of separation. Ultrafiltration centrifugation is to separate free drugs and liposomes by physical interception. During the separation of free drug and liposomes by low-speed centrifugation, choosing the appropriate centrifugation speed and centrifugation time are the key factors for completely separating free TP from liposomes. When optimizing the centrifugation conditions, the overall separation of free drug and blank liposome reached only about 50%. Previous studies found that triptolide had some solubility in pure water and PBS at 25°C, resulting in 17.64 μg/mL and 15.92 μg/mL, respectively. A possible part of the drug dissolved in buffer resulted in a higher sealing rate measured by this method than the actual rate. In the process of separating free drug and liposome by glucan gel column, the drug leakage in the liposome system is diluted by the addition of a large amount of drug adsorption in the gel column, resulting in the consistent elution curve of free drug and liposome solution. Ultrafiltration centrifugation method does not require high equipment and saves time. This method can accurately determine the encapsulation rate of drugs, so the ultrafiltration centrifugation method is finally selected to determine the encapsulation rate of triptolide liposome. Because the ultrafiltration tube has a certain adsorption effect on drugs, pre-saturation treatment

needs to be conducted before ultrafiltration centrifugal operation. In addition, due to the existence of the "concentration polarization" effect, the membrane transmittance of free drugs will be low. Therefore, the liposome to test solution should be diluted in the experimental process to avoid the occurrence of this phenomenon.

S5. Release curve-fitting equations of TP、TP@LIP and TF-TP@LIP.

S5.1 Release curve-fitting equations of TP.

Model	Fitting equation	Correlation coefficient r
First-order release model	$\ln(1\text{-}Q) = -0.7354\ t + 1.5030$	0.9896
Weibull model	$\ln(1/1\text{-}Q) = 0.8577\ \ln t - 0.1169$	0.9529
Ritger-Peppas model	$\ln Q = 0.6460\ \ln t - 0.1014$	0.7946

S5.2 Release curve-fitting equations of TP@LIP.

Model	Fitting equation	Correlation coefficient r
First-order release model	$\ln(1\text{-}Q) = -0.7354\ t + 1.5030$	0.9896
Weibull model	$\ln(1/1\text{-}Q) = 0.7262\ \ln t - 0.0208$	0.9630
Ritger-Peppas model	$\ln Q = 0.5505\ \ln t - 0.0995$	0.7515

S5.3 Release curve-fitting equations of TF-TP@LIP.

Model	Fitting equation	Correlation coefficient r
First-order release model	$\ln(1\text{-}Q) = -0.7061\ t + 1.6900$	0.9850
Weibull model	$\ln(1/1\text{-}Q) = 0.6990\ \ln t - 0.1081$	0.9103
Ritger-Peppas model	$\ln Q = 0.5411\ \ln t - 0.0082$	0.8255
